# Supplementary material for: D-LMBmap: a fully automated deep-learning pipeline for whole-brain profiling of neural circuitry
Source: Nat Methods. 2023 Sep 28;20(10):1593–604. doi: 10.1038/s41592-023-01998-6 (PMC10555838; doi:10.1038/s41592-023-01998-6)
Supplement: Supplementary file 1 — Supplementary Figs. 1–8, Tables 1, 3–5 and all supplementary legends. [file 41592_2023_1998_MOESM1_ESM.pdf]

nature methods

Article https://doi.org/10.1038/s41592-023-01998-6

D-LMBmap: a fully automated deep-learning pipeline for whole-brain profiling of neural circuitry

In the format provided by the
authors and unedited

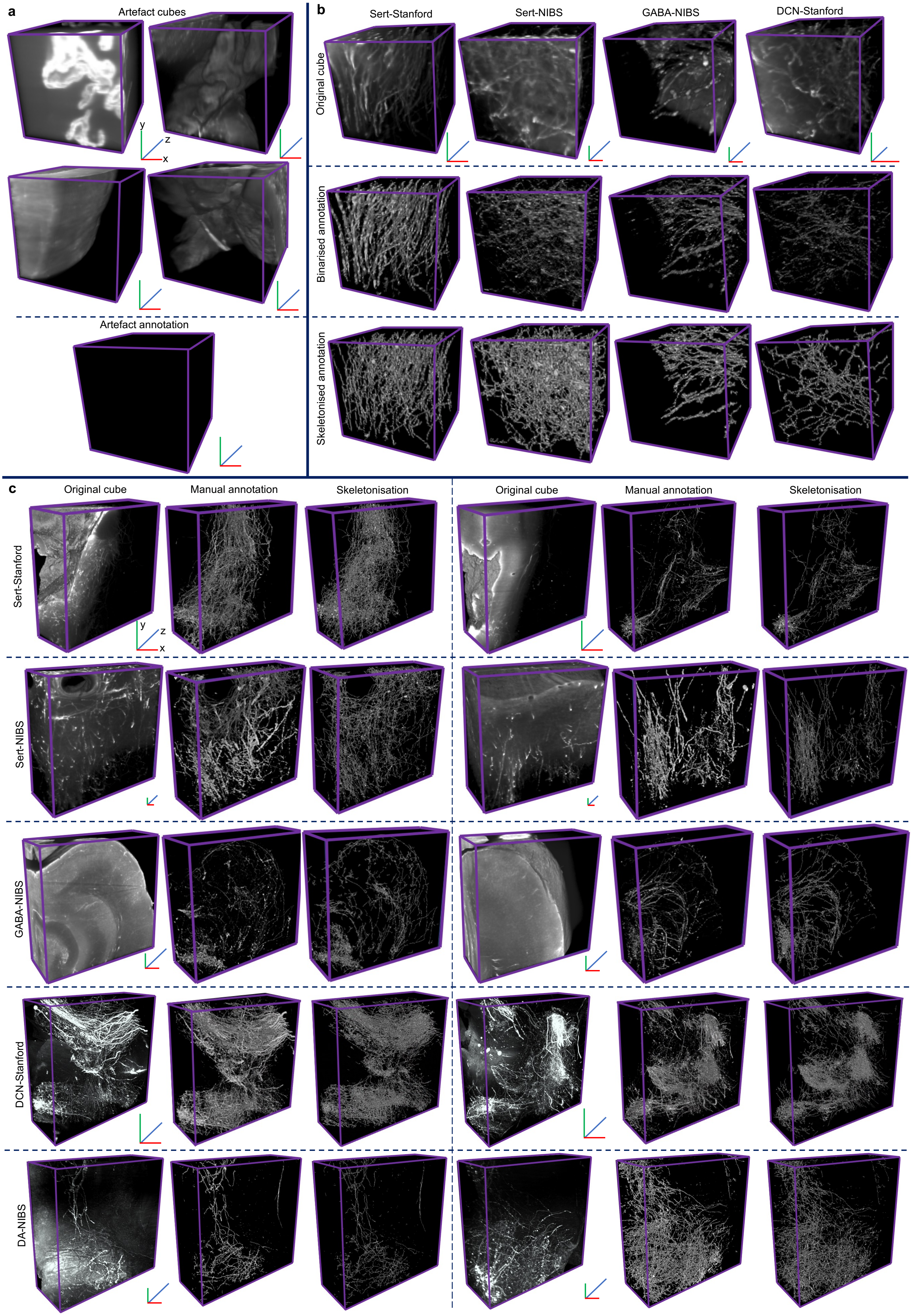

Supplementary Fig.1 | Automatically annotated 3D cubes for training and manual annotated 3D cubes for testing.

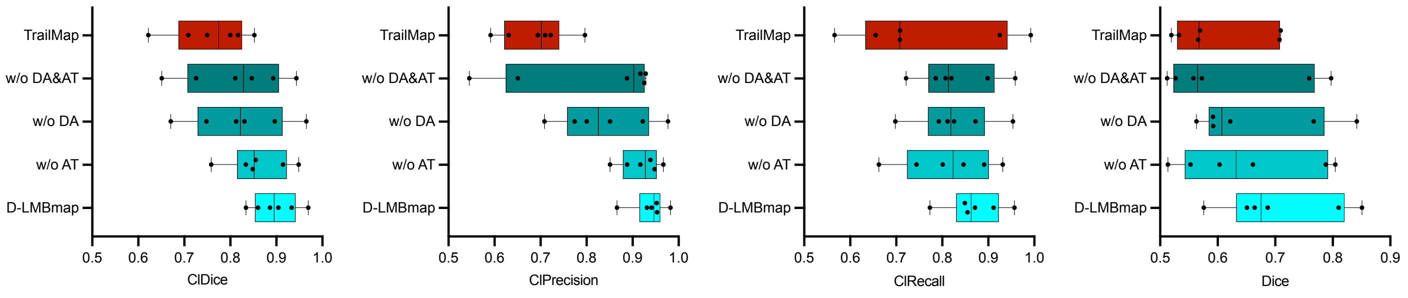

Supplementary Fig. 2 | Ablation study of the modules used in the whole-brain axon segmentation pipeline.

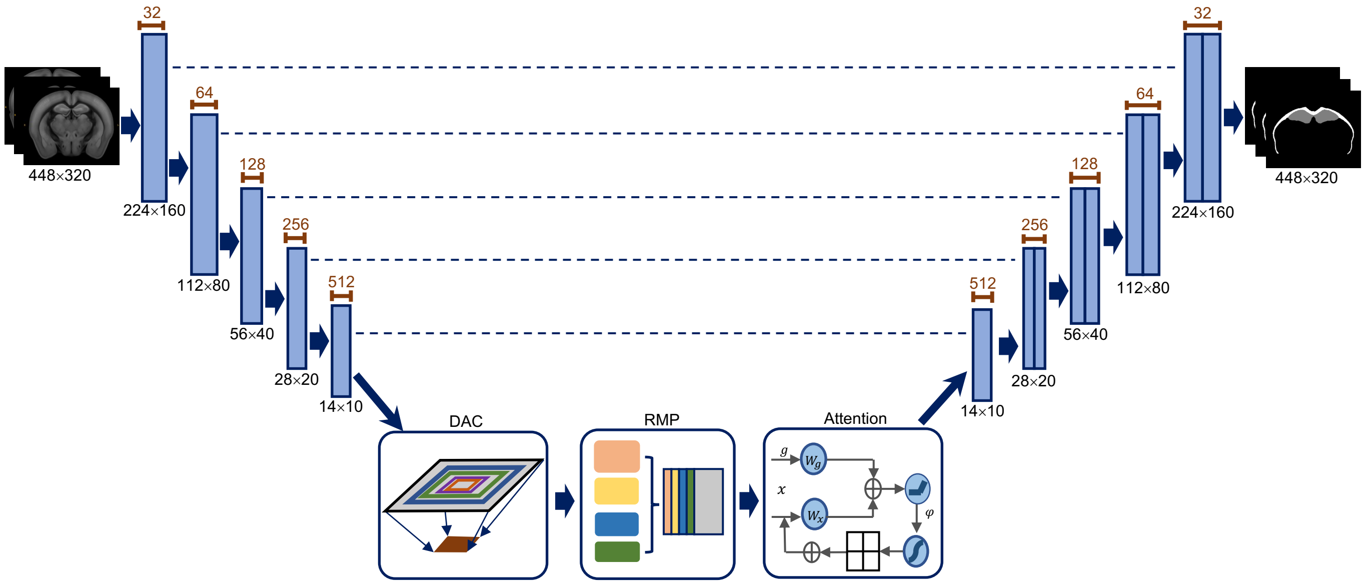

Supplementary Fig. 3 | Style transfer solution employed in D-LMBmap.

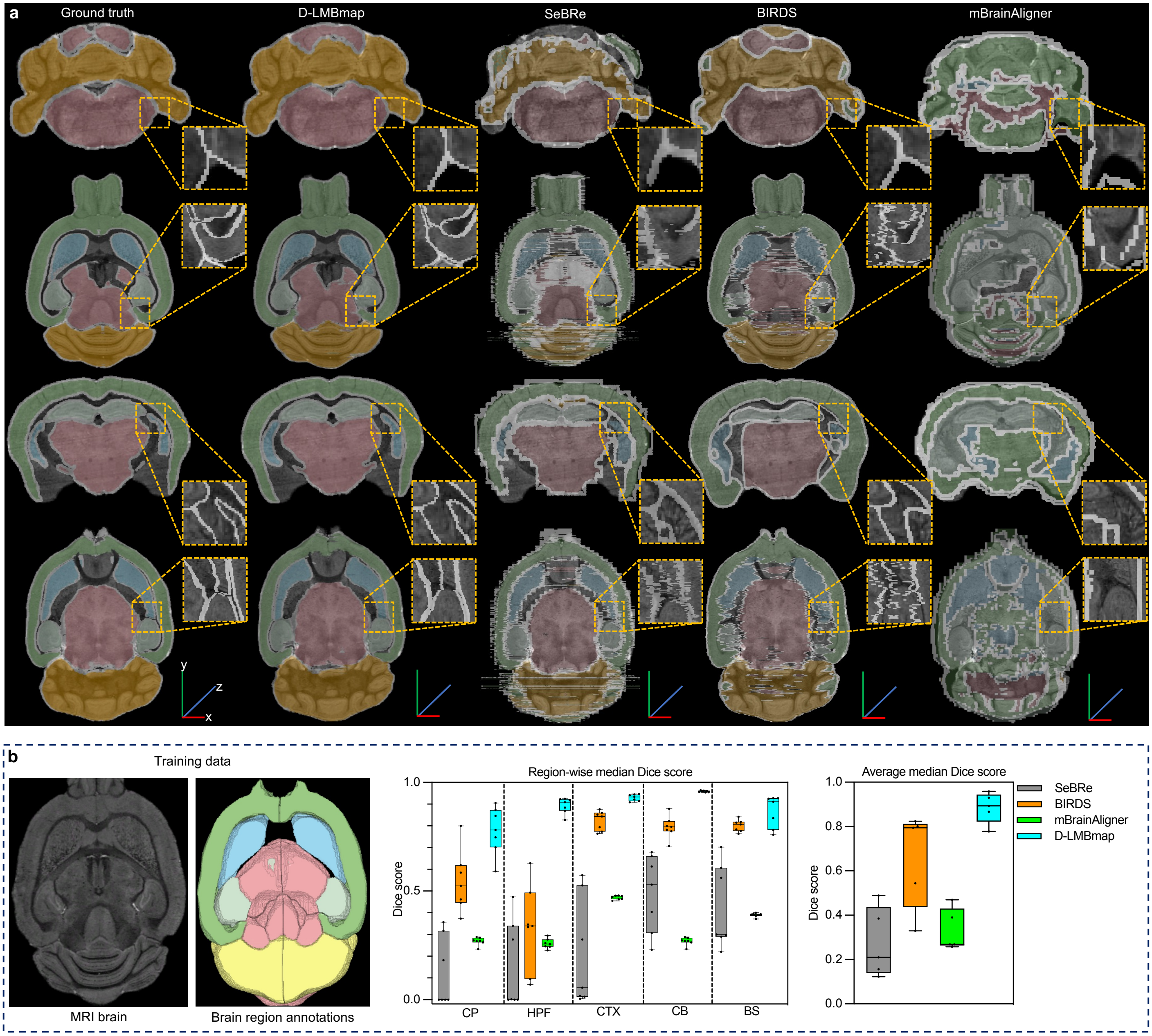

Supplementary Fig. 4 | Comparison of brain region segmentation results of MRI brains by different methods.

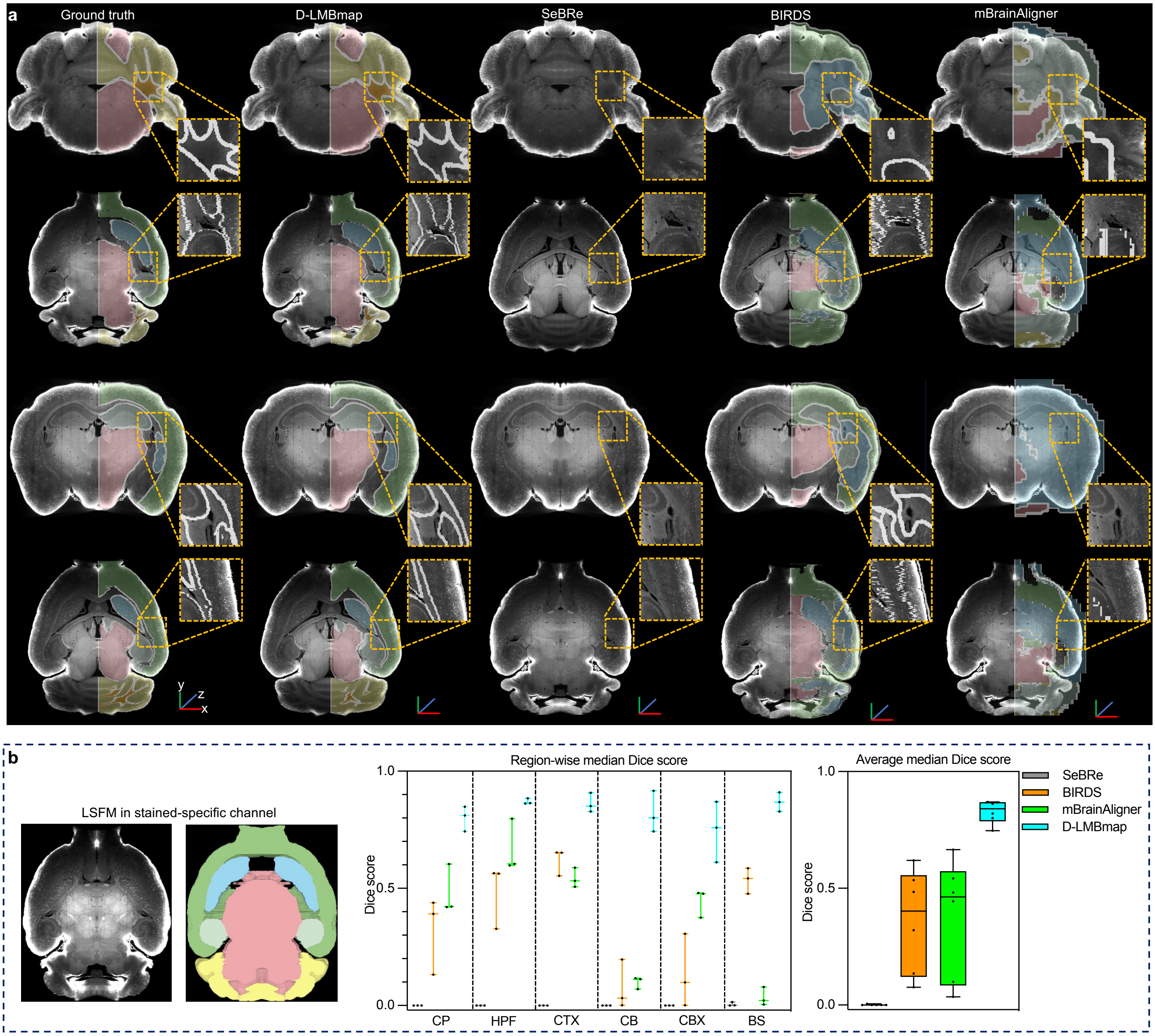

Supplementary Fig. 5 | The comparison of brain region segmentation results of LSFM brains imaged in the stained-specific channel.

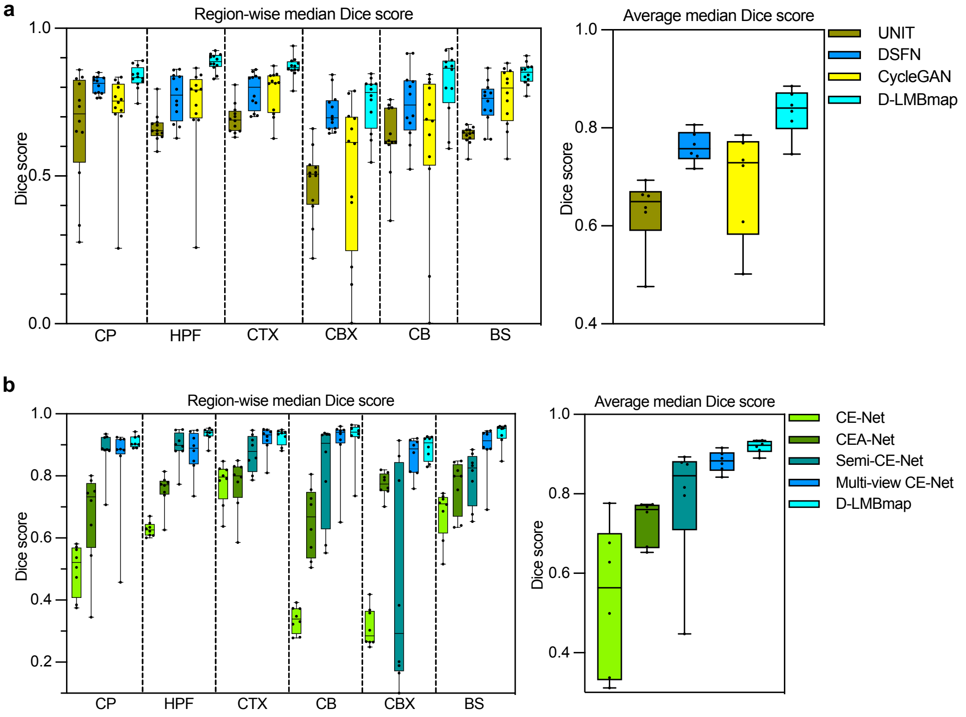

Supplementary Fig. 6 | Ablation study for effectiveness evaluation of the strategies applied in D-LMBmap brain region segmentation module.

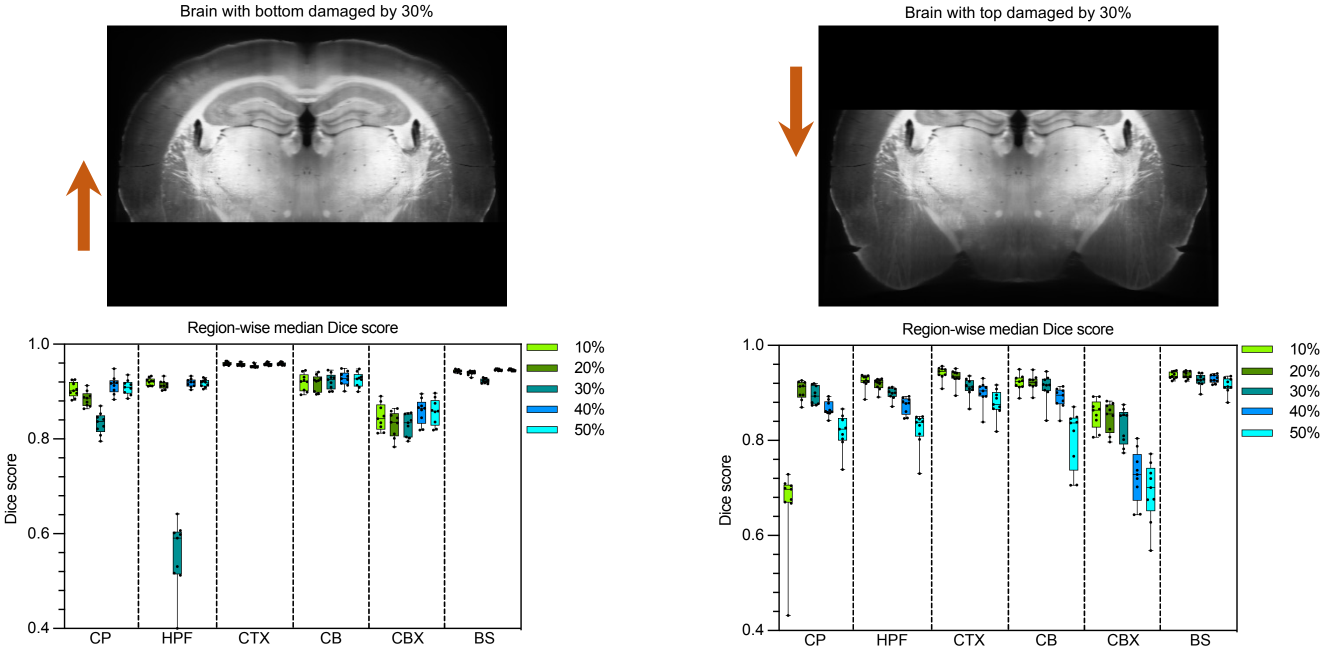

Supplementary Fig. 7 | Quantitative evaluation of registration methods on damaged brain.

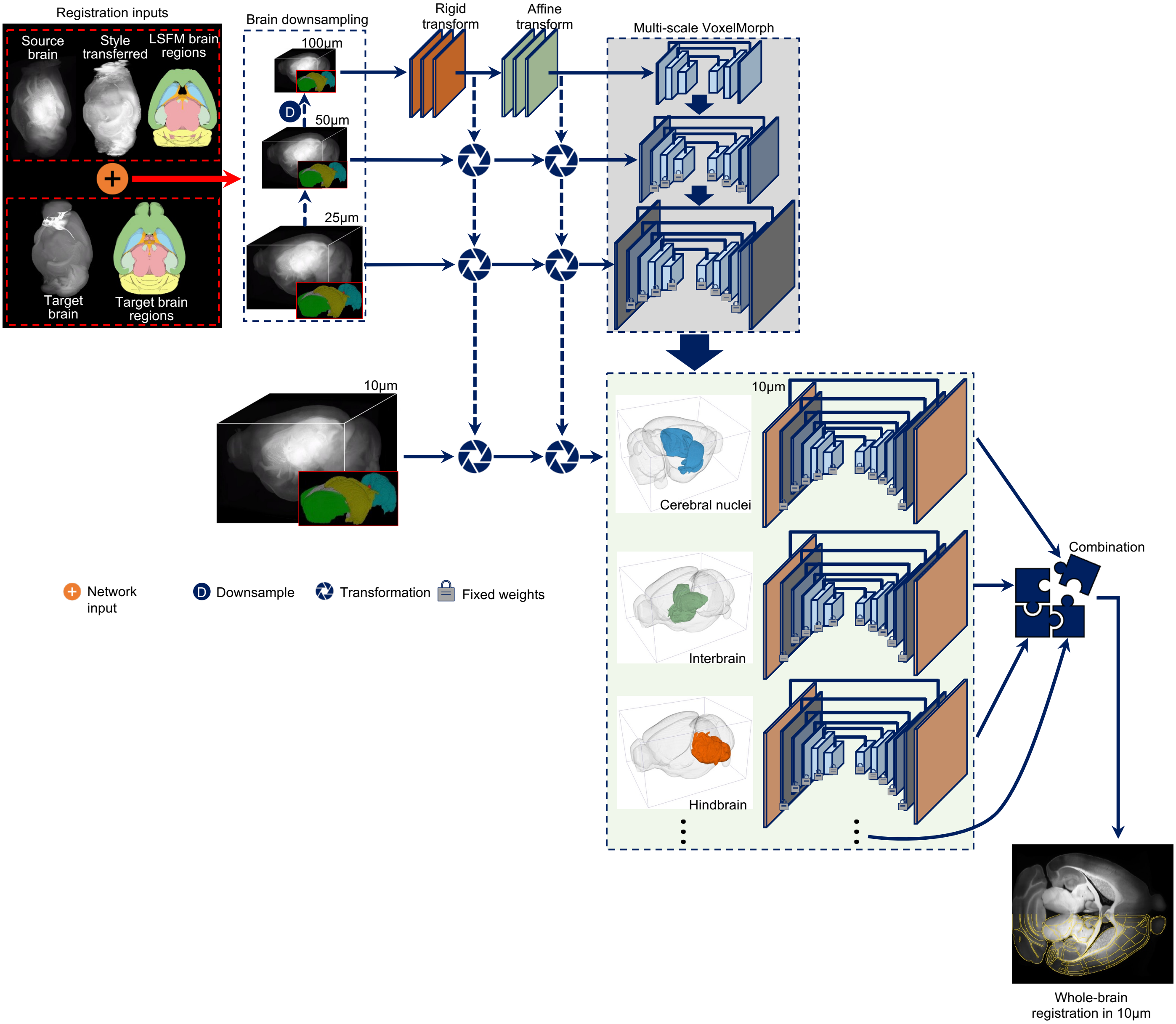

Supplementary Fig. 8 | Extended D-LMBmap pipeline for the whole-brain registration in higher resolution.

Supplementary Table 1. Summary of the samples used.

Modality	Institutes	Tissue Clearing Method	Stained Signals	Imaging plane	Imaging resolution (X, Y, Z in μm)	Whole brain or half brain	Brain name	Tasks
LSFM	LMB	Adipo-Clear	Cell bodies (anti-RFP)	Horizontal	4.0625, 4.0625, 3	Whole brain	Soma-stained	BRS, WBR
			Nuclei (anti-cFos)	Horizontal	4.0625, 4.0625, 3	Whole brain	Nuclei-stained	BRS, WBR
	Stanford University	Adipo-Clear	Axons of Serotonergic Neurons (anti-GFP)	Sagittal	4.0625, 4.0625, 3	Half brain	Sert-Stanford	WBAS, BRS, WBR
			Axons of the DCN (anti-RFP)	Horizontal	4.0625, 4.0625, 3	Whole brain	DCN-Stanford	WBAS
	NIBS	iDISCO	Axons of the Serotonergic Neurons (LINCS)	Horizontal	1.625, 1.625, 3	Half brain	Sert-NIBS	WBAS, BRS, WBR
			Axons of the GABAergic Neurons in the VTA (LINCS)	Horizontal	1.625, 1.625, 3	Whole brain	GABA-NIBS	WBAS
			Axon of the Dopaminergic Neurons (LINCS)	Horizontal	1.625, 1.625, 3	Whole brain	DA-NIBS	WBAS
MRI	UCL	–	FVB_NCrl	Horizontal	4.0625, 8.125, 8	Whole brain	MRI	BRS, WBR

Abbreviation

DCN: Deep cerebellar nuclei

VTA: Ventral tegmental area

BRS: Brain region segmentation

WBR: Whole-brain registration

WBAS: Whole-brain axon segmentation

Supplementary Table. 3-1 Datasets used for whole-brain axon segmentation (WBAS)

Brain name	Whole-brain resolution	No. of brains	Training samples (automated annotation)				Testing samples (manual annotation)	
			No. of axon cubes	No. of artefact cubes	No. of cubes after data augmentation	Cube size	Number of cubes	Cube size
Sert-Stanford	2160×2560×2078	5	46	54	1040	150×150×150	2	600×600×225
DCN-Stanford	2160×2560×1892	16	49	47	725	150×150×150	2	600×600×225
Sert-NIBS	7233×7199×1184	9	86	10	1024	150×150×150	2	600×600×225
GABA-NIBS	3753×3748×997	3	91	45	1452	150×150×150	2	600×600×225
DA-NIBS	3619×3602×1023	1	84	100	1156	150×150×150	2	600×600×225

Supplementary Table. 3-2 Number of cubes used for the evaluation of training samples selected from different brain regions

Brain name	Cubes located brain regions		CP	HPF	CTX	CB	BS	Cube size
DCN-Stanford	No. of cubes for training	“axon” cubes	7	7	7	8	7	150×150×150
		“artefact" cubes	8	8	7	8	6	150×150×150
	No. of cubes for testing	/	3	3	3	3	3	150×150×150

Supplementary Table. 4 Datasets used for brain region segmentation (BRS) and whole-brain registration (WBR)

Brain name	Resized whole-brain resolution	Total No. of brains	Brains with annotations													Training samples for BRS	Testing samples for BRS	Training samples for WBR	Testing samples for WBR
			No. of Brains	Major brain regions							Small brain regions					No. of training brains	No. of testing brains	No. of training brains	No. of testing brains
				BS	CTX	CB	CBX	CP	HPF	LV&3rd V	ACT	FR	MTT	IPN	MH_LH				
Soma-stained	320×456×528	20	3	1063	1183	376	376	530	564	689	227	113	110	237	142	1	2	10	3
Nuclei-stained	320×456×528	12	3	1151	1279	334	424	571	609	623	213	113	96	222	142	1	2		3
Sert-Stanford	320×456×528	5	3	1099	1356	269	269	567	623	632	200	120	105	185	124	1	2		3
Sert-NIBS	320×456×528	9	3	1074	1098	444	452	505	526	/	/	/	/	/	/	1	2		3
MRI	224×288×448	8	8	2112	2473	951	/	941	839	/	/	/	/	/	/	1	7	5	3
Allen CCFv3	320×456×528	1	1	348	416	132	133	155	252	203	100	73	38	40	61	1	/	1	/

Supplementary Table. 5 Required GPU memory, training and testing time of D-LMBmap for whole-brain registration under different imaging resolution. * indicates the values by theoretically computation.

Imaging resolution (voxel)	Brain and region size	Training phase				Testing (registration) phase	
		GPU memory used	No. of training brains	No. of training epochs	Training time	Processor	Registration time per brain
100μm	Whole-brain 80×114×132	9.5G	9	1000	6 hr 42 min	AMD Ryzen 5600X	27 sec
50μm	Whole-brain 160×228×264	10.6G	9	300	5 hr 19 min		2.9 min
25μm	Whole-brain 320×456×528	24G	9	300	49 hr 42 min		8 min
10μm	Whole-brain 800×1140×1320	360.6G*	9	300	495 hr*		46 min*
	Cerebral Cortex (CTX) 735×1032×1038	48.3G*	9	300	20 hr 39 min*		31.7 min*
	Cerebral nuclei (CNU) 498×785×438	16.7G*	9	300	4 hr 48 min*		7.7 min*
	Interbrain (IB) 485×588×458	15.0G*	9	300	3 hr 46 min*		6.1 min*
	Midbrain (MB) 520×508×375	13.6G*	9	300	2 hr 58 min*		4.9 min*
	Hindbrain (HB) 402×608×460	14.2G*	9	300	3 hr 18 min*		5.4 min*
	Cerebellar cortex (CBX) 575×900×328	16.9G*	9	300	4 hr 53 min*		7.8 min*
	Cerebellar nuclei (CBN) 145×560×142	9.8G*	9	300	42 min*		1.5 min*

Supplementary Fig.1 | Automatically annotated 3D cubes for training and manual annotated 3D cubes for testing.

a, Four selected "pure" artefact cubes for deep model training with diverse types of artefacts and their annotations were assigned with no signal. The cube size is 150×150×150 voxels. (Scale bar, X, Y, Z=60μm.)

b, Four selected "pure" axon cubes for deep model training with different types of axons, and their binarised annotations and skeletonised annotations. Cubes are from Sert-Stanford, Sert-NIBS, DCN-Stanford, and GABA-NIBS brains respectively. The cube size is 150×150×150 voxels. (Scale bar, X, Y, Z=60μm.)

c, Ten manually annotated cubes for quantitative evaluation of axon segmentation efficiency on different types of axons. The third and the sixth rows shows their skeletonised manual annotations. Cubes are selected from brain Sert-Stanford, Sert-NIBS, GABA-NIBS, DCN-Stanford, and DA-NIBS. The cube size is 600×600×225 voxels. (Scale bar, X, Y=240μm, Z=90μm.)

Supplementary Fig. 2 | Ablation study of the modules used in the whole-brain axon segmentation pipeline.

ClDice, ClPrecision, ClRecall, and Dice score are used for the evaluation of axon segmentation results generated by D-LMBmap without both data augmentation and axial attention (w/o DA&AT), without data augmentation (w/o DA), and without axial attention (w/o AT). Manually annotated cubes are from the brains of Sert-Stanford, GABA-NIBS, and DA-NIBS, n=6. Box plot: centre line, median; box limits, upper and lower quartiles; whiskers, 1.5× interquartile range; points, individual data points.

Supplementary Fig. 3 | Style transfer solution employed in D-LMBmap.

The deep neural network architecture of the developed CEA-Net, which is a U-Net like structure with three modules added, including dense atrous convolution (DAC), residual multi-kernel pooling (RMP), and attention gate.

Supplementary Fig. 4 | Comparison of brain region segmentation results of MRI brains by different methods.

a, The comparison of brain region segmentation results of an MRI brain among D-LMBmap, SeBRe, BIRDS, and mBrainAligner. (Scale bar, X, Y, Z=1mm).

b, Quantitative evaluation of different brain region segmentation methods in terms of region-wise and average median Dice score on MRI brains (n=7). Only one MRI brain is used for training in the sample-trained pipeline. Left: the brain data and annotations used for training the Multi-view Semi-CEA deep model. Middle: region-wise median Dice score for five brain regions (CP, HPF, CTX, CB, and BS). Right: average median Dice score of different methods. Box plot: centre line, median; box limits, upper and lower quartiles; whiskers, 1.5× interquartile range; points, individual data points.

Supplementary Fig. 5 | The comparison of brain region segmentation results of LSFM brains imaged in the stained-specific channel.

a, Comparisons were made between D-LMBmap, SeBRe, BIRDS, and

mBrainAligner for the brain region segmentation results of an LSFM brain imaged in the stained-specific channel. (Scale bar, X, Y, Z=1mm.)

b, Quantitative evaluation of different brain region segmentation methods in terms of region-wise and average median Dice score on LSFM stained-specific brains (n=3). Allen brain atlas is used for training for all four methods. Left: an LSFM brain in stained-specific channel and the segmented brain regions; Middle: region-wise median Dice scores for six brain regions (CP, HPF, CTX, CB, CBX, and BS); Right: average median Dice scores of different methods. Box plot: centre line, median; box limits, upper and lower quartiles; whiskers, 1.5× interquartile range; points, individual data points.

Supplementary Fig. 6 | Ablation study for effectiveness evaluation of the strategies applied in D-LMBmap brain region segmentation module.

a, Quantitative comparison of four style transfer methods on six major brain regions (CP, HPF, CTX, CBX, CB, BS). The segmentation effectiveness of "Atlas-trained pipeline" when employed with different style transfer methods were evaluated by Dice score (n= 12 brains).

b, Ablation study for testing the effectiveness of the modules applied in brain region segmentation. The segmentation backbone is CE-Net, and the attention gate, semi-supervised learning, and multi-view strategy are the modules we developed, which contribute to the performance of D-LMBmap for brain region segmentation. Region-wise and average median Dice scores are reported from 8 LSFM autofluorescence brains. Left: region-wise median Dice score for six brain regions (CP, HPF, CTX, CB, CBX, and BS). Right: average median Dice score of different methods. Box plot: centre line, median; box limits, upper and lower quartiles; whiskers, 1.5× interquartile range; points, individual data points.

Supplementary Fig. 7 | Quantitative evaluation of registration methods on damaged brain.

The performance of D-LMBmap on the registration of damaged brains. Left: registration results of D-LMBmap when a brain is damaged from top to bottom with the percentage damage ranging from 10% to 50%. Right: registration results of D-LMBmap when a brain is damaged from bottom to top with the percentage damage ranging from 10% to 50% (n=3). Box plot: centre line, median; box limits, upper and lower quartiles; whiskers, 1.5× interquartile range; points, individual data points.

Supplementary Fig. 8 | Extended D-LMBmap pipeline for the whole-brain registration in higher resolution.

The initial registration parameters and deformation space are obtained by the whole-brain registration using 25μm resolution images first by our current pipeline. Each major brain region is further registered to Allen CCFv3 atlas at a resolution of 10um, and integrated together to archive whole-brain registration at higher resolution.

Supplementary Video. 1 | Comparing the performance of TrailMap and D-LMBmap on 3D cubes having different axon densities.

From left to right for each row: an original 3D cube with a size of 200×200×225 voxels, the axon segmentation results by TrailMap, the axon segmentation results by D-LMBmap.

Supplementary Video. 2 | Comparison of TrailMap and D-LMBmap on a representative 3D cube with rotation and zoom.

From left to right, an original 3D cube with a size of 150×150×150 voxels, the axon segmentation results by TrailMap, the axon segmentation results by D-LMBmap.

Supplementary Video. 3 | Whole-brain axon segmentation results of a Sert-Stanford brain.

Supplementary Video. 4 | Flythrough of the results of whole-brain registration and axon density heatmap of a Sert-Stanford brain in horizontal, sagittal, and coronal views.

From top to bottom, the figure shows the results of whole-brain registration and the heatmap of axon density analysis of a GABA-NIBS brain in horizontal, sagittal, and coronal views.

From left to right, it includes a flythrough of the brain imaged in autofluorescence and stained-specific channels; the corresponding sections of the Allen atlas; the corresponding sections of the registered brain in autofluorescence and stained-specific channels, respectively, where the segmented axons are overlaid (in pink) on the stained-specific channel brain; and the corresponding heatmaps of axon density in each brain region after registration to the Allen atlas.

Supplementary Video. 5 | Flythrough of the results of whole-brain registration and axon density heatmap of a Sert-NIBS brain in horizontal, sagittal, and coronal views.

From top to bottom, the figure shows the results of whole-brain registration and the heatmap of axon density analysis of a GABA-NIBS brain in horizontal, sagittal, and coronal views.

From left to right, it includes a flythrough of the brain imaged in autofluorescence and stained-specific channels; the corresponding sections of the Allen atlas; the corresponding sections of the registered brain in autofluorescence and stained-specific channels, respectively, where the segmented axons are overlaid (in pink) on the stained-specific channel brain; and the corresponding heatmaps of axon density in each brain region after registration to the Allen atlas.

Supplementary Video. 6 | Flythrough of the results of whole-brain registration and axon density heatmap of a GABA-NIBS brain in horizontal, sagittal, and coronal views.

From top to bottom, the figure shows the results of whole-brain registration and the heatmap of axon density analysis of a GABA-NIBS brain in horizontal, sagittal, and coronal views.

From left to right, it includes a flythrough of the brain imaged in autofluorescence and stained-specific channels; the corresponding sections of the Allen atlas; the corresponding sections of the registered brain in autofluorescence and stained-specific channels, respectively, where the segmented axons are overlaid (in pink) on the stained-specific channel brain; and the corresponding heatmaps of axon density in each brain region after registration to the Allen atlas.

Supplementary Video. 7 | A step-by-step tutorial on using D-LMBmap's primary Functions.

Supplementary Table. 1 | Summary of the samples used.

Supplementary Table. 2 | Exported average axon density in each brain regions across the whole brain by D-LMBmap. There are five sheets, including Sert-Stanford (n=3), Sert-NIBS (n=3), GABA-NIBS (n=3), DCN-Stanford (n=3), and DA-NIBS (n=1). The brain region taxonomy and hierarchy are based on the Allen atlas.

Supplementary Table. 3-1 | Datasets used for whole-brain axon segmentation.

Supplementary Table. 3-2 | Number of cubes used for the evaluation of training samples selected from different brain regions.

Supplementary Table. 4 | Datasets used for brain region segmentation and whole-brain registration.

Supplementary Table. 5 | Required GPU memory, training and testing time of D-LMBmap for whole-brain registration under different imaging resolution. For the brain imaging resolution in 100μm, 50μm, 25μm, the required GPU memory and training time were practically recorded by everytime training the D-LMBmap registration pipeline from scratch using a computing server with a NVIDIA GeForce RTX 3090 graphics-processing unit. The testing (registration) time in 100μm, 50μm, 25μm were practically recorded by using a laptop with an AMD Ryzen 5600X central processing unit. For the whole brain imaged in 10μm, the required GPU memory, training time (training from scratch) were theoretically computed by training the D-LMBmap registration pipeline from scratch. For major brain regions in 10μm, the required GPU memory and training time were theoretically computed based on initial deformation parameters provided by the registration in 25μm resolution. All testing time for whole-brain and major brain regions in 10μm were theoretically computed by using a laptop with an AMD Ryzen 5600X central processing unit.

* indicates the values by theoretically computation.
