# Supplementary material for: D-LMBmap: a fully automated deep-learning pipeline for whole-brain profiling of neural circuitry
Source: Nat Methods. 2023 Sep 28;20(10):1593–604. doi: 10.1038/s41592-023-01998-6 (PMC10555838; doi:10.1038/s41592-023-01998-6)
Supplement: Supplementary file 2 — Reporting Summary [file 41592_2023_1998_MOESM2_ESM.pdf]

nature portfolio

Corresponding author(s): Jing Ren

Last updated by author(s): Jul 6, 2023

Reporting Summary

Nature Portfolio wishes to improve the reproducibility of the work that we publish. This form provides structure for consistency and transparency in reporting. For further information on Nature Portfolio policies, see our Editorial Policies and the Editorial Policy Checklist.

Statistics

For all statistical analyses, confirm that the following items are present in the figure legend, table legend, main text, or Methods section.

n/a	Confirmed	
☐	☒	The exact sample size (*n*) for each experimental group/condition, given as a discrete number and unit of measurement
☐	☒	A statement on whether measurements were taken from distinct samples or whether the same sample was measured repeatedly
☐	☒	The statistical test(s) used AND whether they are one- or two-sided<br>*Only common tests should be described solely by name; describe more complex techniques in the Methods section.*
☒	☐	A description of all covariates tested
☐	☒	A description of any assumptions or corrections, such as tests of normality and adjustment for multiple comparisons
☐	☒	A full description of the statistical parameters including central tendency (e.g. means) or other basic estimates (e.g. regression coefficient) AND variation (e.g. standard deviation) or associated estimates of uncertainty (e.g. confidence intervals)
☐	☒	For null hypothesis testing, the test statistic (e.g. *F*, *t*, *r*) with confidence intervals, effect sizes, degrees of freedom and *P* value noted<br>*Give P values as exact values whenever suitable.*
☒	☐	For Bayesian analysis, information on the choice of priors and Markov chain Monte Carlo settings
☒	☐	For hierarchical and complex designs, identification of the appropriate level for tests and full reporting of outcomes
☒	☐	Estimates of effect sizes (e.g. Cohen's *d*, Pearson's *r*), indicating how they were calculated

Our web collection on statistics for biologists contains articles on many of the points above.

Software and code

Policy information about availability of computer code

Data collection	We use imageJ (Fiji) (version 1.53q) to select manual annotated cubes. ITK-SNAP (version 3.6.0) was used for manual annotation of brain images.
Data analysis	All the softwares used in this study are listed below as "Software, Developer, Link".<br>PyTorch 1.11.0, The Linux Foundation, https://pytorch.org<br>PyCharm 2022.3, JetBrains, https://www.jetbrains.com/<br>Anaconda 4.12.0, Anaconda Inc, https://www.anaconda.com/<br>Python 3.8.8, Python Software Foundation, https://www.python.org/<br>ImageJ (Fiji) 1.53q, National Institutes of Health, https://imagej.nih.gov/ij/index.html<br>Elastix 4.8, Image Sciences Institute, https://elastix.lumc.nl/<br>IMARIS 9.0.1, Oxford Instruments, https://imaris.oxinst.com/<br>Vaa3D 4.001, Allen Institute, https://alleninstitute.org/what-we-do/brain-science/research/products-tools/vaa3d/<br>ITK-SNAP 3.6.0, Paul A. Yushkevich et al. 2006, www.itksnap.org<br>Allen Institute's Common Coordinate Framework (CCFv3), Allen Institute's for Brain Science, http://atlas.brain-map.org<br>GraphPad Prism 9.4.1, GraphPad, https://www.graphpad.com/

For manuscripts utilizing custom algorithms or software that are central to the research but not yet described in published literature, software must be made available to editors and reviewers. We strongly encourage code deposition in a community repository (e.g. GitHub). See the Nature Portfolio guidelines for submitting code & software for further information.

Data

Policy information about availability of data

All manuscripts must include a data availability statement. This statement should provide the following information, where applicable:

- Accession codes, unique identifiers, or web links for publicly available datasets
- A description of any restrictions on data availability
- For clinical datasets or third party data, please ensure that the statement adheres to our policy

The datasets generated and analysed in this study are available at the D-LMBmap's Github page (https://github.com/lmbneuron/D-LMBmap). All the automatically annotated and manually annotated samples are also available at the Github page. All the source data files for each figure are available at https://doi.org/10.5281/zenodo.8123585. The full resolution LSFM brain images are available on request. All the MRI brain data are available at https://github.com/dmac-lab/mouse-brain-atlas. The Allen Institute's Common Coordinate Framework (CCFv3) atlas are available at http://atlas.brain-map.org.

Human research participants

Policy information about studies involving human research participants and Sex and Gender in Research.

Reporting on sex and gender	N/A
Population characteristics	N/A
Recruitment	N/A
Ethics oversight	N/A

Note that full information on the approval of the study protocol must also be provided in the manuscript.

Field-specific reporting

Please select the one below that is the best fit for your research. If you are not sure, read the appropriate sections before making your selection.

☒ Life sciences ☐ Behavioural & social sciences ☐ Ecological, evolutionary & environmental sciences

For a reference copy of the document with all sections, see nature.com/documents/nr-reporting-summary-flat.pdf

Life sciences study design

All studies must disclose on these points even when the disclosure is negative.

Sample size	34 LSFM mouse brains imaged in 647 channel with 25 manually annotated cubes were used for the evaluation of whole-brain axon segmentation. 54 LSFM brains imaged in 488 channel with 12 brains, 30302 image slices containing manual annotated brain regions were used for the evaluation of brain region segmentation and whole-brain registration. This is so far all the diverse data collected and manual annotated in our project. It's sufficient to lead to a determination that systematic errors surpassed statistical errors.
Data exclusions	No data were excluded from the analyses.
Replication	We successfully tested against 25 cubes on axon segmentation, 12 brains on brain style transfer, brain region segmentation, and whole-brain registration.
Randomization	Randomization was not performed as multiple experimental groups across biological samples were not used in this study.
Blinding	All annotators worked in isolation and were blinded to group allocation.

Reporting for specific materials, systems and methods

We require information from authors about some types of materials, experimental systems and methods used in many studies. Here, indicate whether each material, system or method listed is relevant to your study. If you are not sure if a list item applies to your research, read the appropriate section before selecting a response.

Materials & experimental systems

n/a	Involved in the study	
☐	☒	Antibodies
☒	☐	Eukaryotic cell lines
☒	☐	Palaeontology and archaeology
☐	☒	Animals and other organisms
☒	☐	Clinical data
☒	☐	Dual use research of concern

Methods

n/a	Involved in the study	
☒	☐	ChIP-seq
☒	☐	Flow cytometry
☒	☐	MRI-based neuroimaging

Antibodies

Antibodies used	Rabbit anti-Fos (Synaptic Systems, CAT 226003, lot 9-95; dilution: 1:500 for iDISCO), rabbit anti-RFP (Rockland, 600-401-379, lot 42896, dilution:1:500), donkey anti-rabbit AlexaFluor 647 (Jackson ImmunoResearch, 711-605-152, lot: 161533; dilution: 1:500)
Validation	Fos: synthetic peptide corresponding to rat Fos AA2-17, website states specific to Fos, validated for immunohistochemistry in mouse tissue on the website and in several publications e.g. DeNardo et al. Nature Neuroscience, 2019. RFP: website states antibody is expected to cross-react with RFP variants (e.g. mCherry, tdTomato) and has been validated for immunohistochemistry in mouse in several publications (e.g. Crowther et al, Stem Cell Reports, 2018) and for iDISCO in mouse tissue (DeNardo et al. Nature Neuroscience, 2019)

Animals and other research organisms

Policy information about studies involving animals; ARRIVE guidelines recommended for reporting animal research, and Sex and Gender in Research

Laboratory animals	TRAP2;Ai14 mice (TRAP2, JAX 03032; Ai14, JAX 7914) were kindly shared by Liqun Luo, Stanford University, CA, US. DAT-Cre (JAX 006660) and Vgat-Cre mice (JAX 028862) were obtained from the Jackson Laboratory. Sert-Cre mice (031028-UCD) were obtained from the University of California, Davis. All the mice used in this study are adult mice (8-16 weeks old).
Wild animals	This study did not involve wild animals.
Reporting on sex	The sex of each mouse is irrelevant to our study, which focused on algorithm-development for whole-brain 3D image analysis.
Field-collected samples	This study did not involve field-collected samples
Ethics oversight	All experiments related to the use of mice in the Medical Research Council Laboratory of Molecular Biology (MRC LMB) were carried out in accordance with the UK Animals (Scientific Procedures) Act of 1986, with local ethical approval provided by the MRC LMB Animal Welfare Ethical Review Board (LMB AWERB) and overseen institutionally by designated animal welfare officers (NACWOs). Animal Project Licence (PPL) is PP6471806. All experiments related to the use of mice at the National Institute of Biological Sciences, Beijing (NIBS) were approved by the Animal Care and Use Committee of NIBS in accordance with the Regulations for the Administration of Affairs Concerning Experimental Animals of China.

Note that full information on the approval of the study protocol must also be provided in the manuscript.
